# Association between Obesity Indices and Insulin Resistance among Healthy Korean Adolescents: The JS High School Study

**DOI:** 10.1371/journal.pone.0125238

**Published:** 2015-05-13

**Authors:** Sun Min Lim, Dong Phil Choi, Yumie Rhee, Hyeon Chang Kim

**Affiliations:** 1 Department of Internal Medicine, Yonsei University College of Medicine, Seoul, Korea; 2 Department of Preventive Medicine, Yonsei University College of Medicine, Seoul, Korea; 3 Severance Institute for Vascular and Metabolic Research, Yonsei University College of Medicine, Seoul, Korea; 4 Cardiovascular and Metabolic Diseases Etiology Research Center, Yonsei University College of Medicine, Seoul, Korea; Virgen Macarena University Hospital, School of Medicine, University of Seville, SPAIN

## Abstract

**Objective:**

To investigate whether indices of obesity are associated with insulin resistance in Korean adolescents.

**Methods:**

This study was conducted as a cross-sectional analysis of 817 healthy adolescents aged 15–16 years without diabetes. Percentiles group of weight-for-height, body mass index (BMI)-for-age, waist circumference (WC)-for-age, and skin fold thickness (SFT)-for-age were based on the 2007 Korean National Growth Charts. Percentiles of waist-to-hip ratio (WHR), waist-to-height ratio (WHtR), and percent body fat were calculated for the study population. Insulin resistance was estimated by homeostatic model assessment (HOMA-IR). Logistic regression models were used to estimate odds ratio for insulin resistance according to seven obesity indices. Generalized linear models were used to assess the associations between obesity indices and continuous HOMA-IR levels.

**Results:**

Sex and age-adjusted odds ratios (95% confidence interval) for insulin resistance, defined as HOMA-IR>2.50, of the 75–94th and ≥95th percentiles of weight-for-height were 3.87 (2.38–6.30) and 11.37 (5.87–22.02), compared to the <50th percentile. Corresponding odds ratios were 3.27 (2.02–5.28) and 11.72 (6.05–22.73) for BMI-for-age, 4.72 (2.82–7.88) and 13.22 (6.42–27.23) for WC-for-age, 3.67 (2.27–5.94) and 13.58 (6.71–27.48) for WHR, 4.78 (2.99–7.67) and 12.84 (6.23–26.46) for WHtR, 2.62 (1.61–4.26) and 6.68 (3.46–12.90) for SFT-for-age, and 2.29 (1.33–4.26) and 10.06 (4.39–23.06) for body fat. These associations were more prominent when insulin resistance was defined as HOMA-IR>3.16 and were stronger in males than in females. Continuous measure of HOMA-IR was significantly associated with body weight, BMI, WC, WHR, WHtR, and SFT in both sexes (p<0.001), and with percent body fat in males only (p<0.001).

**Conclusion:**

Our findings suggest that obesity indices are positively associated with insulin resistance in apparently healthy adolescents.

## Introduction

Obesity is a growing concern worldwide. The prevalence of childhood obesity has more than doubled in the last two decades [1]. The epidemic status of obesity is associated with an increasing number of children and adolescents with type 2 diabetes [2]. Overweight and obesity are important risk factors of type 2 diabetes; they have been shown to be associated with insulin resistance in persons with normal glucose level [3]. As well, an inverse linear association between body mass index (BMI) and age at diagnosis of type 2 diabetes has been reported [4], spurring interest as to whether abnormal glucose metabolism can be detected at an early age. In previous studies increased risk for insulin resistance was noted for adolescents with prominent obesity [5, 6]. The association between obesity and insulin resistance was also reported in Korean patients with hyperlipidemia and middle-aged Korean offspring of hypertensive parents [7, 8]. Meanwhile, data on whether being overweight is associated with insulin resistance in healthy adolescents are lacking. In this study, we investigated the association between various indices of obesity and insulin resistance as measured by HOMA-IR in a healthy Korean adolescent population.

## Materials and Methods

### Study participants

This study was conducted as a cross-sectional analysis of baseline data collected for cohort study. Health examinations and questionnaire were administered to all first-year students at a rural high school throughout April 2007, June 2010, and June 2011. Of the 852 available first-year students (287 in 2007, 282 in 2010, and 283 in 2011), 817 students (278 in 2007, 275 in 2010, and 264 in 2011) aged 15–16 years who completed all physical examinations, blood laboratory tests, and the self-reported questionnaire were enrolled in the study. All participants had no previously diagnosis of diabetes mellitus or hypertension. Written informed consent was obtained from each participant and his/her parent or guardian. Informed consent forms were distributed to eligible students at least one week prior to the examination, allowing the participating students and their parents enough time to understand the purpose and procedures of the study. On the day of examination, research staff checked whether each consent form was completed and signed by the student, as well as his/her parent or guardian. The study protocol and consent procedure was approved by the Institutional Review Board of Severance Hospital at Yonsei University College of Medicine (Approval No. 4–20100169).

### Measurements

Individual health-related lifestyles, as well as personal and family disease history, were evaluated with self-administered questionnaires. Anthropometric measurements were performed according to a predefined protocol. Standing height was measured to the nearest 0.1 cm on a stadiometer. Body weight was measured to the nearest 0.1 kg on a digital scale, with the subject wearing their school uniform. Body mass index (BMI) was calculated as weight divided by the square of height (kg/m^2^). Waist circumference (WC) was measured to the nearest 0.1 cm at the level of the superior iliac crest at the end of a normal expiration. Hip circumference was measured at the level of widest circumference over the greater trochanters. Waist-to-hip ratio (WHR) was calculated as waist circumference divided by hip circumference. Waist-to-height ratio (WHtR) was calculated as waist circumference divided by height. Each triceps and subscapular skin fold thickness (SFT) was measured twice, and the average of the four measurements was used for analysis. Percent body fat was measured by bioelectrical impedance analysis in only 277 students in 2007 (GIF-891DX, Gil Woo, Korea) and in 264 students in 2011 (InBody 720, Biospace, Korea).

Systolic and diastolic blood pressures (SBP and DBP) were measured on the right arm with an oscillometric device (Dinamap 1846 SX/P, USA). Two readings at 5 min interval were obtained and averaged to determine SBP and DBP for each individual. If the two readings differed by more than 10 mmHg, additional readings were obtained and the last two readings were averaged. Smoking (male: n = 6 [1.4%] and female: n = 1 [0.3%]) and drinking (male: n = 14 [3.3%] and female: n = 9 [2.3%]) histories were obtained by self-reported questionnaires; however, they were not included in the multivariate analysis, as the small number of participants would not have affected the final outcomes.

Fasting blood samples were drawn after at least an 8-hour fast. Serum concentrations of total cholesterol, high-density lipoprotein (HDL) cholesterol, triglycerides, aspartate aminotransferase (AST), and alanine aminotransferase (ALT) were measured by enzymatic methods with an autoanalyzer. Fasting glucose level was measured by a glucose hexokinase method. Insulin level was measured on the basis of a radio-immunometric method. Insulin resistance was assessed by the homeostatic model assessment (HOMA-IR), calculated as the product of the fasting plasma insulin level (IU/l) and the fasting plasma glucose level (mmol/l) divided by 22.5. Insulin resistance is generally defined as a HOMA-IR >2.50 in adults [9], but a recent study suggested a higher cut-off point >3.16 for children and adolescents [10]; thus we defined insulin resistance using these two different cutpoints.

### Statistical analysis

Sex-specific percentile values of weight-for-height, BMI-for-age, WC-for-age, and SFT-for-age were obtained from the 2007 Korean National Growth Charts [11, 12]. Sex-specific percentile values of WHR, WHtR, and percent body fat were based on their distribution in the study population, because they were not available in the growth chart. Participants were classified into four groups according to percentiles (<50^th^, 50–74^th^, 75–94^th^, and ≥95^th^ percentiles) according to the seven obesity indices. Distributions of cardiovascular and metabolic risk factors were determined for these four groups, and their linear trends were tested. Associations between weight-for-height percentiles and metabolic abnormalities were assessed using logistic regression models. Metabolic abnormalities in these analyses included high blood pressure (SBP ≥120 mmHg or DBP ≥80 mmHg), high total cholesterol (≥200 mg/dl), high triglycerides (≥150mg/dl), elevated liver enzymes (AST ≥30 IU/l or ALT ≥30 IU/l), high fasting glucose (≥100 mg/dl), and high HOMA-IR (>2.50 and >3.16). Odds ratios and 95% confidence interval (CI) for each metabolic abnormality were obtained for the 50–74^th^, 75–94^th^, and ≥95 percentiles groups, compared to the lowest (<50^th^ percentile) weight-for-height group. Sex and age-adjusted odds ratios (95% CI) for insulin resistance were also estimated according to the percentiles for the other obesity indices (BMI-for-age, WC-for-age, WHR, WHtR, SFT-for-age, and percent body fat). Insulin resistance was defined using two different cut-offs for HOMA-IR: >2.50 and >3.16. Obesity indices were analysed as categorical variables (<50^th^, 50–74^th^, 75–94^th^, and ≥95 percentiles) as well as continuous variables (per one standard deviation increase). Finally, associations of the seven obesity indices and HOMA-IR levels were assessed using generalized linear models separately for males and females after adjustment for age. Means of HOMA-IR were calculated by the least-squares method for four percentile groups of weight-for-height, BMI-for-age, WC-for-age, WHR, WHtR, SFT-for-age, and percent body fat. Incremental HOMA-IR value was also calculated per one SD increase of body weight, BMI, WC, WHR, WHtR, SFT, and percent body fat. All statistical analyses were two-tailed and performed using SAS software version 9.1 (SAS Institute, Cary, NC, USA). All *P*-values <0.05 ware considered statistically significant.

## Results

The characteristics of the 817 (418 male and 399 female) participants are shown in Table 1. Although study participants were recruited from a single high school, their anthropometric and blood pressure distributions were similar to those in the 2007 Korean National Growth Charts. The median height was 171.4 /159.5 cm (male/female) in this study, and the corresponding value from the growth charts was 170.3/159.6 cm. The median body weight was 63.4/53.3 kg (male/female) in this study and 60.1/53.1 kg in the growth charts. None of the adolescents had a fasting glucose higher than 110 mg/dl. Only seven adolescents (0.9%) were current smokers, and 23 (2.8%) consumed alcoholic drinks at least once a month.

**Table 1:** Characteristics of Study Participants.

Variables	Total (n = 817)	Male (n = 418)	Female (n = 399)	*p*
Age, year	15.8 ± 0.3	15.8 ± 0.3	15.8 ± 0.3	0.989
Height, cm	165.7 ± 7.8	171.4 ± 5.4	159.7 ± 5.0	<.001
Weight, kg	59.7 ± 10.8	65.1 ± 10.8	54.0 ± 7.3	<.001
BMI, kg/m^2^	21.7 ± 3.0	22.1 ± 3.3	21.1 ± 2.6	<.001
WC, cm	73.6 ± 7.7	75.3 ± 8.4	71.9 ± 6.6	<.001
WHR	0.78 ± 0.05	0.80 ± 0.05	0.77 ± 0.04	<.001
WHtR	0.44 ± 0.04	0.44 ± 0.05	0.45 ± 0.04	<.001
SFT, cm	16.1 ± 6.2	13.5 ± 5.7	18.9 ± 5.4	<.001
Percent body fat*	21.4 ± 7.7	16.6 ± 6.3	26.4 ± 5.5	<.001
SBP, mmHg	108 ± 12	113 ± 12	103 ± 11	<.001
DBP, mmHg	59 ± 7	60 ± 7	59 ± 7	0.421
Total cholesterol, mg/dl	154 ± 26	148 ± 26	161 ± 25	<.001
HDL cholesterol, mg/dl	44 ± 9	41 ± 8	47 ± 9	<.001
Triglycerides, mg/dl	81 ± 29	83 ± 31	78 ± 27	0.015
Total/HDL-cholesterol ratio	3.59 ± 0.77	3.68 ± 0.82	3.49 ± 0.71	<.001
AST, IU/l	20 ± 6	22 ± 4	18 ± 3	<.001
ALT, IU/l	16 ± 10	19 ± 12	13 ± 5	<.001
Fasting glucose, mg/dl	88 ± 7	90 ± 7	87 ± 7	<.001
Fasting insulin, uIU/mL	8.7 ± 3.2	8.7 ± 3.4	8.7 ± 2.9	0.867
HOMA-IR	1.91 ± 0.79	1.94 ± 0.86	1.87 ± 0.70	0.229
Weight-for-height ≥95 percentile	51 (6.2%)	26 (6.2%)	25 (6.3%)	0.367
BMI-for-age ≥95 percentile	50 (6.1%)	28 (6.7%)	22 (5.5%)	0.464
WC-for-age ≥95 percentile	45 (5.5%)	15 (3.6%)	30 (7.5%)	<.001
SFT-for-age ≥95 percentile	53 (6.5%)	18 (4.3%)	35 (8.8%)	0.067
WHR ≥95 percentile	42 (5.1%)	21 (5.0%)	21 (5.3%)	0.999
WHtR ≥95 percentile	39 (4.8%)	20 (4.8%)	19 (4.8%)	0.999
Percent body fat ≥95 percentile	29 (5.4%)	15 (5.4%)	14 (5.3%)	0.999
Ever smoker ≥100 cigarette	7 (0.9%)	6 (1.4%)	1 (0.3%)	0.145
Alcohol intake ≥1/month	23 (2.8%)	14 (3.3%)	9 (2.3%)	0.345

Abbreviations: BMI, body mass index; WC, waist circumference; WHR, waist-to-hip ratio; WHtR, waist-to-height ratio; SFT, skin-fold thickness; SBP, systolic blood pressure; DBP, diastolic blood pressure; AST, aspartate aminotransferase; ALT, alanine aminotransferase; HOMA-IR, Homeostasis model assessment insulin resistance.

Data are expressed as mean ± SD or number (%)

*Percent body fat was measured in 541 adolescents (277 males and 264 females).

According to an increase in weight-for-height percentile, body weight, BMI, WC, WHR, WHtR, SFT, body fat, SBP, triglyceride, total/HDL-cholesterol ratio, ALT, fasting glucose, fasting insulin, and HOMA-IR levels increased, while HDL-cholesterol level decreased. Age, height, DBP, total cholesterol, and AST levels were not significantly associated with weight-for-height percentile groups (Table 2).

**Table 2:** Metabolic Characteristics by Weight-for-Height Percentile.

Variable	Mean (95% CI) by Weight-for-Height percentile group				
	<50 percentile (N = 363)	50–74 percentile (N = 228)	75–94 percentile (N = 175)	≥95 percentile (N = 51)	*p* for trend*
Age, year	15.8 (15.8–15.8)	15.8 (15.8–15.9)	15.8 (15.8–15.9)	15.8 (15.7–15.9)	0.546
Height, cm	166.3 (165.6–167.1)	165.1 (164.1–166.1)	165.3 (164.0–166.6)	165.0 (162.5–167.6)	0.087
Weight, kg	53.5 (52.9–54.2)	59.5 (58.5–60.4)	67.2 (65.7–68.6)	78.8 (75.1–82.5)	<.001
BMI, kg/m^2^	19.3 (19.2–19.4)	21.7 (21.6–21.8)	24.4 (24.2–24.7)	28.7 (28.1–29.4)	<.001
WC, cm	68.7 (68.2–69.1)	73.1 (72.6–73.7)	79.9 (79.0–80.8)	89.8 (87.5–92.1)	<.001
WHR	0.77 (0.76–0.77)	0.78 (0.77–0.78)	0.81 (0.81–0.82)	0.86 (0.84–0.88)	<.001
WHtR	0.41 (0.41–0.42)	0.44 (0.44–0.45)	0.48(0.48–0.49)	0.54 (0.53–0.55)	<.001
SFT, cm	12.8 (12.4–13.3)	16.3 (15.7–16.9)	19.4 (18.7–20.2)	27.3 (25.3–29.3)	<.001
Percent body fat**	17.9 (17.1–18.7)	21.8 (20.7–22.8)	25.4 (24.1–26.6)	32.9 (30.7–35.0)	<.001
SBP, mmHg	105 (104–106)	108 (107–110)	112 (110–114)	117 (113–121)	<.001
DBP, mmHg	59 (59–60)	59 (58–60)	60 (59–61)	61 (59–63)	0.158
Total cholesterol, mg/dl	152 (150–155)	156 (152–159)	155 (151–159)	160 (152–168)	0.065
HDL cholesterol, mg/dl	45 (44–46)	45 (44–47)	42 (41–43)	39.6 (37–42)	<.001
Triglycerides, mg/dl	77 (75–80)	79 (76–83)	87 (82–92)	95 (85–105)	<.001
Total/HDL-cholesterol	3.44 (3.37–3.51)	3.52 (3.42–3.62)	3.81 (3.69–3.93)	4.19 (3.88–4.49)	<.001
AST, IU/l	20 (20–21)	20 (19–20)	19 (19–20)	23 (19–27)	0.207
ALT, IU/l	15 (14–15)	15 (15–16)	17 (16–18)	29 (21–36)	<.001
Fasting glucose, mg/dl	88 (87–89)	88 (87–89)	89 (88–90)	90 (88–92)	0.031
Fasting insulin, uIU/mL	7.75 (7.52–7.98)	8.41 (8.08–8.74)	9.88 (9.27–10.48)	12.25 (11.09–13.41)	<.001
HOMA-IR	1.70 (1.64–1.76)	1.84 (1.76–1.92)	2.19 (2.03–2.35)	2.74 (2.46–3.03)	<.001

Abbreviations: BMI, body mass index; WC, waist circumference; WHR, waist-to-hip ratio; WHtR, waist-to-height ratio; SFT, skin-fold thickness; SBP, systolic blood pressure; DBP, diastolic blood pressure; AST, aspartate aminotransferase; ALT, alanine aminotransferase; HOMA-IR, Homeostasis model assessment insulin resistance.

*P-values are adjusted for sex and age (except for the trend of age itself).

**Percent body fat was measured in 541 adolescents (277 males and 264 females).

When compared to adolescents with lower weight-for-height (<50^th^ percentile), those in the 50–74^th^ percentiles exhibited an increased risk for only high blood pressure (p = 0.003). Adolescents in the 75–94^th^ weight-for-height percentile were at increased risks for high blood pressure (p<0.001), hypertriglyceridemia (p = 0.029), and insulin resistance (p<0.001). Adolescents in the ≥95^th^ weight-for-height percentile showed increased risks for high blood pressure (p<0.001), hypercholesterolemia (p = 0.042), hypertriglyceridemia (p = 0.002), elevated liver enzyme (p<0.001), and insulin resistance (p<0.001) (Table 3).

**Table 3:** Risk for Metabolic Abnormalities by Weight-for-Height Percentile.

Dependent variables (metabolic abnormality)	Odds ratio (95% CI) by Weight-for-Height percentile group			
	<50 percentile	50–74 percentile	75–94 percentile	≥95 percentile
Unadjusted				
SBP/DBP ≥120/80 mmHg	1.00	**2.04 (1.18–3.54)**	**4.35 (2.58–7.36)**	**7.70 (3.85–15.40)**
Total cholesterol ≥200 mg/dl	1.00	1.31 (0.62–2.78)	1.18 (0.51–2.72)	**2.89 (1.08–7.77)**
Triglycerides ≥150 mg/dl	1.00	1.94 (0.58–6.42)	**3.43 (1.11–10.64)**	**7.79 (2.17–27.92)**
AST or ALT ≥30 IU/l	1.00	0.91 (0.37–2.20)	1.84 (0.83–4.06)	**7.67 (3.31–17.75)**
Fasting glucose ≥100 mg/dl	1.00	1.11 (0.47–2.63)	1.63 (0.70–3.80)	1.68 (0.46–6.12)
HOMA-IR >2.50	1.00	1.36 (0.80–2.30)	**3.76 (2.32–6.10)**	**10.88 (5.66–20.92)**
HOMA-IR >3.16	1.00	2.14 (0.48–9.66)	**14.62 (4.26–50.11)**	**50.00 (13.82–180.9)**
Adjusted for sex and age				
SBP/DBP ≥120/80 mmHg	1.00	**2.40 (1.36–4.24)**	**5.11 (2.95–8.85)**	**10.08 (4.75–21.39)**
Total cholesterol ≥200 mg/dl	1.00	1.20 (0.56–2.56)	1.13 (0.49–2.63)	**2.83 (1.04–7.71)**
Triglycerides ≥150 mg/dl	1.00	2.06 (0.62–6.86)	**3.56 (1.14–11.08)**	**8.07 (2.23–29.22)**
AST or ALT ≥30 IU/l	1.00	1.02 (0.42–2.49)	1.96 (0.88–4.39)	**9.37 (3.86–22.75)**
Fasting glucose ≥100 mg/dl	1.00	1.21 (0.50–2.89)	1.69 (0.72–3.95)	1.74 (0.47–6.39)
HOMA-IR >2.50	1.00	1.39 (0.81–2.36)	**3.87 (2.38–6.30)**	**11.37 (5.87–22.02)**
HOMA-IR >3.16	1.00	2.20 (0.49–9.94)	**14.80 (4.31–50.76)**	**51.01 (14.07–185.0)**

Abbreviations: SBP, systolic blood pressure; DBP, diastolic blood pressure; AST, aspartate aminotransferase; ALT, alanine aminotransferase; HOMA-IR, Homeostasis model assessment insulin resistance.

All seven indices of obesity had significant positive associations with insulin resistance. These associations were consistent in analyses with categorical variables based on the growth charts percentiles and in analyses with continuous scales of weight-for-height, BMI-for-age, WC-for-age, WHR, WHtR, SFT-for-age, and percent body fat. Among the seven obesity indices, waist circumference (both in percentile groups and in continuous measure) showed the strongest association with risk for having insulin resistance (Table 4).

**Table 4:** Risk for Insulin Resistance by Different Obesity Indices.

Independent variable (obesity index)		HOMA-IR >2.50		HOMA-IR >3.16	
	No. of people	No.	Odds ratio (95% CI)*	No.	Odds ratio (95% CI) *
Weight-for-height percentile					
<50	363	34	1.00	3	1.00
50–74	228	28	1.39 (0.81–2.36)	4	2.20 (0.49–9.94)
75–94	175	49	**3.87 (2.38–6.30)**	19	**14.80 (4.31–50.76)**
≥95	51	27	**11.37 (5.87–22.02)**	15	**51.01 (14.07–185.0)**
BMI-for-age percentile					
<50	360	36	1.00	4	1.00
50–74	225	26	1.18 (0.69–2.01)	5	2.02 (0.54–7.62)
75–94	182	48	**3.27 (2.02–5.28)**	16	**8.48 (2.79–25.78)**
≥95	50	28	**11.72 (6.05–22.73)**	16	**41.52 (13.13–131.3)**
WC-for-age percentile					
<50	333	28	1.00	2	1.00
50–74	255	34	**1.73 (1.02–2.95)**	7	**5.07 (1.04–24.65)**
75–94	184	53	**4.72 (2.82–7.88)**	21	**25.85 (5.93–112.75)**
≥95	45	23	**13.22 (6.42–27.23)**	11	**70.01 (14.54–337.0)**
WHR percentile					
<50	407	38	1.00	5	1.00
50–74	205	30	**1.68 (1.01–2.81)**	8	**3.25 (1.05–10.06)**
75–94	163	45	**3.67 (2.27–5.94)**	17	**9.51 (3.44–26.28)**
≥95	42	25	**13.58 (6.71–27.48)**	11	**30.92 (9.95–96.04)**
WHtR percentile					
<50	408	38	1.00	2	1.00
50–74	205	25	1.39 (0.81–2.37)	8	**8.25 (1.74–39.25)**
75–94	165	53	**4.78 (2.99–7.67)**	20	**28.04 (6.47–121.49)**
≥95	39	22	**12.84 (6.23 + 26. 46)**	11	**80.45 (16.97–381.29)**
SFT-for-age percentile					
<50	354	39	1.00	5	1.00
50–74	238	35	1.49 (0.91–2.45)	8	2.49 (0.80–7.72)
75–94	171	41	**2.62 (1.61–4.26)**	17	**7.88 (2.85–21.77)**
≥95	53	22	**6.68 (3.46–12.90)**	11	**21.04 (6.82–64.88)**
Percent body fat percentile**					
<50	266	37	1.00	7	1.00
50–74	136	29	1.66 (0.97–2.84)	6	1.73 (0.57–5.27)
75–94	110	30	**2.29 (1.33–3.96)**	10	**3.76 (1.39–10.18)**
≥95	29	18	**10.06 (4.39–23.06)**	10	**20.06 (6.83–58.95)**
One SD increase in					
Body weight	817	138	**1.93 (1.61–2.32)**	41	**2.74 (2.06–3.64)**
BMI	817	138	**2.14 (1.78–2.57)**	41	**3.08 (2.30–4.13)**
WC	817	138	**2.23 (1.85–2.68)**	41	**3.29 (2.45–4.44)**
WHR	817	138	**2.16 (1.78–2.63)**	41	**2.98 (2.18–4.08)**
WHtR	817	138	**2.31 (1.91–2.80)**	41	**3.31 (2.46–4.46)**
SFT	816	137	**1.81 (1.52–2.16)**	41	**2.45 (1.88–3.18)**
Percent body fat	541	114	**1.59 (1.29–1.95)**	33	**2.15 (1.56–2.96)**

Abbreviations: BMI, body mass index; WC, waist circumference; WHR, waist-to-hip ratio; WHtR, waist-to-height ratio; SFT, skin-fold thickness; HOMA-IR, Homeostasis model assessment insulin resistance.

*Odds ratios were adjusted for sex and age.

**Percent body fat was measured in 541 adolescents (277 males and 264 females).

**Fig 1:**
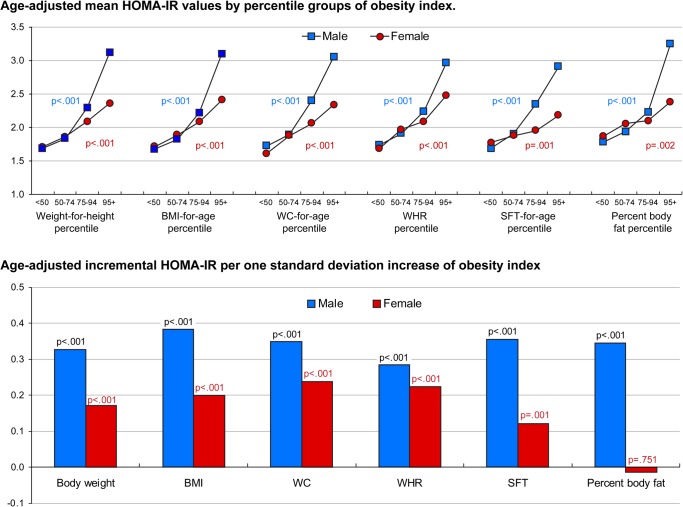
HOMA-IR Levels according to Different Obesity Indices in Male and Female Adolescents. Abbreviations: HOMA-IR, Homeostasis model assessment insulin resistance; BMI, body mass index; WC, waist circumference; WHR, waist-to-hip ratio; WHtR, waist-to-height ratio; SFT, skin-fold thickness. Percent body fat was measured for 541 adolescents (277 males and 264 females). Upper panel shows age-adjusted mean HOMA-IR values by percentile groups for weight-for-height, BMI-for-age, WC-for-age, WHR, WHtR, SFT-for-age, and percent body fat in male and female adolescents. Lower panel shows age-adjusted incremental HOMA-IR per one standard deviation increase in body weight, BMI, WC, WHR, WHtR, SFT and percent body fat in male and female adolescents.

Fig 1 displays the association between obesity indices and HOMA-IR separately for male and female adolescents. In males, mean HOMA-IR levels were strongly and positively associated with percentile groups of weight-for-height, BMI-for-age, WC-for-age, WHR, WHtR, SFT-for-age, and percent body fat (p<0.001 for all). Similar significant associations were observed also in females, although the strength of the associations was weaker than in males. Incremental HOMA-IR values related to one standard deviation increase of obesity indices was the highest for BMI (β = 0.38; p<0.001) and WHtR (β = 0.37; p<0.001) in male adolescents. In females adolescents, WHtR (β = 0.26; p<0.001), WC (β = 0.24; p<0.001), and WHR (β = 022; p<0.001) were associated with incremental increases in HOMA-IR. However, continuous measure of percent body fat was not associated with HOMA-IR in female adolescents (β = −0.01; p = 0.751). Raw data used to create Fig 1 can be found in S1 and S2 Tables. More results from sex-specific analysis are also available in S3 and S4 Tables.

## Discussion

We observed that insulin resistance (measured by HOMA-IR), which is known to increase the risk of developing diabetes, is closely associated with obesity indices in apparently healthy adolescents. All seven indices of obesity (weight, BMI, WC, WHR, WHtR, SFT, body fat) exhibited significant positive associations with insulin resistance. A stronger association between HOMA-IR levels and obesity indices was noted in males than in females.

Insulin resistance accompanying obesity is known to play a key role in diabetes development. In the Bogalusa Heart Study, adverse levels and acceleration of metabolic risk variables, adiposity, and HOMA-IR during childhood and adolescents were closely associated with later onset of diabetes [13]. Interestingly, obesity beginning in childhood has been shown to not only influence the onset of type 2 diabetes but also be the most consistent predictor of adverse changes leading to diabetes, regardless of age, race, or sex. The mechanisms of developing impaired glucose regulation and diabetes may be similar between adults and adolescents; however, it is uncertain when and which factors contribute to insulin resistance in adolescents as different metabolic risk factors can attribute to the development of insulin resistance in different age groups. In a prospective study of both children and adolescents, the most significant predictor of diabetes was WC in 5- to 9-year-olds; 2-hour glucose, BMI and HbA1c in 10- to 14–year-olds; and 2-hour glucose, WC and HbA1c in 15- to 19-year-old subjects [14]. Our findings showed that seven obesity indices are positively associated with insulin resistance even in apparently healthy adolescents, and that even a moderate increase (75–94^th^ percentiles) in obesity indices may be associated with risk of insulin resistance.

In this study, a sex-difference was discovered in the associations between obesity indices and insulin resistance. Mean HOMA-IR levels showed positive progressive associations with obesity indices in male adolescents. However, HOMA-IR values were not associated with percent body fat in female adolescents. These findings suggest that simple anthropometric measurements of general obesity, such as weight-for-height, BMI or SFT, and central obesity markers, such as WC, WHR, or WHtR, can predict obesity-related insulin resistance in female adolescents better than skin fold thickness measurement or bioimpedance analysis. However, hormonal status during puberty, especially for female adolescents, might affect the relationship between obesity and glucose metabolism. In a study of obese children and adolescents, insulin resistance was more common in obese adolescents at higher Tanner stages than in those at lower Tanner stages [15]. Insulin resistance during puberty is related to increases in growth hormone levels due to increasing lipolysis and free fatty acid concentrations. The growth hormone/insulin-like growth factor-I axis contributes to the transient physiological rise in insulin resistance during normal puberty [16, 17]. The growth hormone/insulin-like growth factor-I axis and insulin resistance are involved in the mechanism of adrenarche during prepuberty [18], and insulin resistance reaches its peak during mid-puberty [19]. Insulin sensitivity during normal pre-pubertal and pubertal development differs between males and females. Although the reasons for the sex-differences remain unclear, there are three distinctive differences between males and females that influence the association with insulin resistance; First, females are ahead of males in their physical and sexual development and reach Tanner stages IV-V earlier than males. Second, body fat mass and body fat distribution differ between male and female adolescents [20]. Third, sex hormones during puberty such as oestrogen may influence insulin resistance; a rise in oestrogen level in females may enhance insulin sensitivity due to its role in suppressing secretion of glucagons and protecting against pancreatic insulin responses to glucose [21]. These differences in development and hormonal status between males and females can explain, at least in part, the noted sex difference in the associations between obesity indices and insulin resistance.

Associations between obesity indices and insulin resistance or type 2 diabetes are established in adult populations [3, 22]. Our study showed that various obesity indices are strongly and progressively associated with insulin resistance in a healthy Korean adolescent population. As this study population includes non-diabetic adolescents, as well as very few smokers and alcohol drinkers, it would be unlikely that the association between obesity and insulin resistance would be confounded by other factors. Insulin resistance is generally defined as a HOMA-IR >2.50 in adults, although a cut-off is not established for children and adolescents. In this study we used two different cut-offs (HOMA-IR >2.50 and >3.16). Although the nature of associations between obesity and insulin resistance are similar for both cut-off points, the odds ratio was greater when we used the higher cut-off (HOMA-IR >3.16). This finding is consistent with a previous study suggesting a higher cut-off >3.16 for children and adolescents [10]. In our study population, adolescents with higher obesity indices had markedly increased odds for having insulin resistance, although their fasting glucose levels were normal and not associated with obesity indices. This finding is partially in line with previous studies in which anthropometric indexes were not found to be associated with fasting glucose levels in children and adolescents [23, 24].

This study has several limitations. First, insulin resistance was not measured by direct methods, such as glucose clamp or frequently sampled intravenous glucose tolerance test. However, HOMA-IR has been reported to be a reliable measure of insulin resistance in children and adolescents [9, 25]. Second, this study comprised a relatively small sample size of which the participants were homogenous. Thus, it may not be large enough to detect differences in the association of various obesity indices or differences between males and females. Third, most study participants were in the midst of puberty, a period when hormonal status may influence the indices of obesity, as well as insulin sensitivity. Female adolescents enter puberty earlier and have more fat mass, and these differences can act as confounding factors when comparing sexes. Moreover, physiological insulin resistance during puberty cannot be distinguished from insulin resistance arising from obesity [26].

In conclusion, this study suggests that moderate overweight or obesity is positively associated with higher insulin resistance in healthy adolescents. Simple anthropometric measurements and their comparison to standard growth charts can identify adolescents at higher risk for insulin resistance or diabetes. Regular screening with anthropometric measurements at schools should be enforced, as adolescents would be expected to benefit from screening strategies and early intervention. Our findings suggested that HOMA-IR, which is a validated surrogate marker of insulin resistance, should be used instead of fasting plasma glucose level to identify high-risk adolescents and to prevent them from developing diabetes. Further prospective studies are required to evaluate whether weight control in adolescents actually improves insulin sensitivity and prevents the onset of diabetes.

## Supporting Information

S1 Table Age-adjusted mean HOMA-IR values by percentile groups of obesity index. (DOCX)

S2 Table Age-adjusted incremental HOMA-IR per one standard deviation increase of obesity index. (DOCX)

S3 Table Metabolic characteristics according to weight-for-height percentile in male and female adolescents. (DOCX)

S4 Table Risk for insulin resistance according to different obesity indices in male and female adolescents. (DOCX)
