# Supplementary material for: Association between Obesity Indices and Insulin Resistance among Healthy Korean Adolescents: The JS High School Study
Source: PLoS One. 2015 May 13;10(5):e0125238. doi: 10.1371/journal.pone.0125238 (PMC4429969; doi:10.1371/journal.pone.0125238)
Supplement: S1 Table — (DOCX) [file pone.0125238.s001.docx]

S1 Table. Age-adjusted mean HOMA-IR values by percentile groups of obesity index

| Obesity index percentile groups | | Mean HOMA-IR | |
| --- | --- | --- | --- |
|  |  | Male | Female |
| Weight-for-height percentile | <50 | 1.68 | 1.71 |
|  | 50-74 | 1.83 | 1.86 |
|  | 75-94 | 2.29 | 2.08 |
|  | ≥95 | 3.12 | 2.36 |
| BMI-for-age percentile | <50 | 1.67 | 1.71 |
|  | 50-74 | 1.82 | 1.88 |
|  | 75-94 | 2.22 | 2.08 |
|  | ≥95 | 3.10 | 2.41 |
| WC-for-age percentile | <50 | 1.72 | 1.61 |
|  | 50-74 | 1.89 | 1.88 |
|  | 75-94 | 2.40 | 2.06 |
|  | ≥95 | 3.05 | 2.34 |
| WHR percentile | <50 | 1.73 | 1.68 |
|  | 50-74 | 1.90 | 1.96 |
|  | 75-94 | 2.24 | 2.09 |
|  | ≥95 | 2.96 | 2.48 |
| WHtR percentile | <50 | 1.69 | 1.67 |
|  | 50-74 | 1.86 | 1.91 |
|  | 75-94 | 2.37 | 2.21 |
|  | ≥95 | 3.07 | 2.44 |
| SFT-for-age percentile | <50 | 1.68 | 1.77 |
|  | 50-74 | 1.90 | 1.87 |
|  | 75-94 | 2.34 | 1.95 |
|  | ≥95 | 2.91 | 2.18 |
| Percent body fat percentile | <50 | 1.77 | 1.86 |
|  | 50-74 | 1.93 | 2.05 |
|  | 75-94 | 2.23 | 2.10 |
|  | ≥95 | 3.25 | 2.38 |

Abbreviations: HOMA-IR, Homeostasis model assessment insulin resistance; BMI, body mass index; WC, waist circumference; WHR, waist-to-hip ratio; WHtR, waist-to-height ratio; SFT, skin-fold thickness.
