# Supplementary material for: Association between Obesity Indices and Insulin Resistance among Healthy Korean Adolescents: The JS High School Study
Source: PLoS One. 2015 May 13;10(5):e0125238. doi: 10.1371/journal.pone.0125238 (PMC4429969; doi:10.1371/journal.pone.0125238)
Supplement: S2 Table — (DOCX) [file pone.0125238.s002.docx]

S2 Table. Age-adjusted incremental HOMA-IR per one standard deviation increase of obesity index.

| One standard deviation increase of obesity index | Incremental HOMA-IR | |
| --- | --- | --- |
|  | Male | Female |
| Body weight | 0.33 | 0.17 |
| BMI | 0.38 | 0.20 |
| WC | 0.35 | 0.24 |
| WHR | 0.29 | 0.22 |
| WHtR | 0.37 | 0.26 |
| SFT | 0.36 | 0.12 |
| Percent body fat | 0.35 | -0.01 |

Abbreviations: BMI, body mass index; WC, waist circumference; WHR, waist-to-hip ratio; WHtR, waist-to-height ratio; SFT, skin-fold thickness; SBP, systolic blood pressure; DBP, diastolic blood pressure; AST, aspartate aminotransferase; ALT, alanine aminotransferase; HOMA-IR, Homeostasis model assessment insulin resistance.
