# Supplementary material for: Association between Obesity Indices and Insulin Resistance among Healthy Korean Adolescents: The JS High School Study
Source: PLoS One. 2015 May 13;10(5):e0125238. doi: 10.1371/journal.pone.0125238 (PMC4429969; doi:10.1371/journal.pone.0125238)
Supplement: S3 Table — (DOCX) [file pone.0125238.s003.docx]

S3 Table. Metabolic Characteristics by Weight-for-Height Percentile in Male and Female Adolescents

| Variable | Mean (95% CI) by Weight-for-Height percentile group | | | | p for trend* |
| --- | --- | --- | --- | --- | --- |
|  | <50 percentile | 50-74 percentile | 75-94 percentile | ≥95 percentile |  |
| **Male** | N=196 | N=106 | N=90 | N=26 |  |
| Age, year | 15.8 ± 0.3 | 15.8 ± 0.3 | 15.8 ± 0.3 | 15.8 ± 0.3 | 0.544 |
| Height, cm | 171.3 ± 5.3 | 171.1 ± 5.6 | 171.7 ± 5.4 | 171.8 ± 6.1 | 0.453 |
| Weight, kg | 57.6 ± 5.6 | 65.2 ± 5.3 | 74.6 ± 6.5 | 88.9 ± 9.0 | <.001 |
| BMI, kg/m^2^ | 19.6 ± 1.2 | 22.2 ± 0.8 | 25.3 ± 1.3 | 30.0 ± 2.2 | <.001 |
| WC, cm | 69.6 ± 4.0 | 75.0 ± 3.8 | 82.6 ± 5.5 | 94.7 ± 6.6 | <.001 |
| WHR | 0.78 ± 0.04 | 0.79 ± 0.04 | 0.83 ± 0.04 | 0.89 ± 0.05 | <.001 |
| WHtR | 0.41 ± 0.02 | 0.44 ± 0.02 | 0.48 ± 0.03 | 0.55 ± 0.04 | <.001 |
| SFT, cm | 10.1 ± 2.6 | 13.6 ± 3.5 | 16.8 ± 4.5 | 26.4 ± 8.2 | <.001 |
| Percent body fat** | 13.2 ± 3.7 | 17.0 ± 4.5 | 20.6 ± 5.6 | 31.7 ± 5.2 | <.001 |
| SBP, mmHg | 109 ± 10 | 113 ± 12 | 119 ± 12 | 125 ± 10 | <.001 |
| DBP, mmHg | 59 ± 7 | 59 ± 7 | 60 ± 7 | 63 ± 7 | 0.010 |
| Total cholesterol, mg/dl | 145.9 ± 23.0 | 147.3 ± 24.8 | 149.5 ± 29.1 | 157.7 ± 31.1 | 0.037 |
| HDL cholesterol, mg/dl | 43 ± 9 | 42 ± 8 | 38 ± 7 | 37 ± 6 | <.001 |
| Triglycerides, mg/dl | 77 ± 29 | 83 ± 31 | 92 ± 32 | 100 ± 36 | <.001 |
| Total/HDL-cholesterol | 3.5 ± 0.7 | 3.6 ± 0.8 | 4.0 ± 0.8 | 4.4 ± 1.2 | <.001 |
| AST, IU/l | 22 ± 4 | 21 ± 6 | 21 ± 4 | 28 ± 18 | 0.034 |
| ALT, IU/l | 17 ± 6 | 18 ± 6 | 21 ± 8 | 40 ± 35 | <.001 |
| Fasting glucose, mg/dl | 89 ± 7 | 90 ± 7 | 90 ± 7 | 92 ± 7 | 0.010 |
| Fasting insulin, uIU/mL | 7.6 ± 2.1 | 8.2 ± 2.1 | 10.2 ± 4.6 | 13.7 ± 4.5 | <.001 |
| HOMA-IR | 1.7 ± 0.5 | 1.8 ± 0.5 | 2.3 ± 1.2 | 3.1 ± 1.1 | <.001 |
| **Female** | N=167 | N=122 | N=85 | N=25 |  |
| Age, year | 15.8 ± 0.3 | 15.8 ± 0.3 | 15.8 ± 0.3 | 15.8 ± 0.4 | 0.130 |
| Height, cm | 160.5 ± 4.5 | 159.9 ± 5.1 | 158.5 ± 5.4 | 158.0 ± 5.6 | 0.001 |
| Weight, kg | 48.8 ± 4.0 | 54.4 ± 4.3 | 59.3 ± 6.0 | 68.3 ± 7.1 | <.001 |
| BMI, kg/m2 | 18.9 ± 1.1 | 21.2 ± 0.7 | 23.5 ± 1.1 | 27.3 ± 1.5 | <.001 |
| WC, cm | 67.6 ± 4.2 | 71.5 ± 3.8 | 77.1 ± 4.8 | 84.7 ± 6.4 | <.001 |
| WHR | 0.75 ± 0.04 | 0.76 ± 0.04 | 0.79 ± 0.04 | 0.83 ± 0.06 | <.001 |
| WHtR | 0.42 ± 0.02 | 0.45 ± 0.02 | 0.49 ± 0.02 | 0.54 ± 0.03 | <.001 |
| SFT, cm | 16.0 ± 4.3 | 18.6 ± 3.7 | 22.2 ± 4.4 | 28.3 ± 5.7 | <.001 |
| Percent body fat** | 23.5 ± 4.0 | 26.7 ± 4.6 | 29.4 ± 5.2 | 34.0 ± 6.0 | <.001 |
| SBP, mmHg | 100 ± 11 | 104 ± 10 | 104 ± 9 | 108 ± 11 | <.001 |
| DBP, mmHg | 59 ± 7 | 59 ± 6 | 59 ± 7 | 58 ± 10 | 0.482 |
| Total cholesterol, mg/dl | 159.7 ± 26.4 | 162.7 ± 25.1 | 160.4 ± 23.6 | 162.6 ± 23.7 | 0.724 |
| HDL cholesterol, mg/dl | 48 ± 9 | 49 ± 10 | 46 ± 9 | 43 ± 9 | 0.004 |
| Triglycerides, mg/dl | 77 ± 24 | 75 ± 25 | 82 ± 31 | 90 ± 33 | 0.024 |
| Total/HDL-cholesterol | 3.4 ± 0.7 | 3.5 ± 0.7 | 3.6 ± 0.7 | 3.9 ± 0.9 | <.001 |
| AST, IU/l | 18 ± 3 | 18 ± 3 | 17 ± 4 | 18 ± 3 | 0.096 |
| ALT, IU/l | 13 ± 3 | 13 ± 5 | 13 ± 5 | 17 ± 11 | 0.009 |
| Fasting glucose, mg/dl | 87 ± 7 | 87 ± 7 | 87 ± 6 | 88 ± 6 | 0.725 |
| Fasting insulin, uIU/mL | 7.9 ± 2.4 | 8.6 ± 2.8 | 9.6 ± 3.4 | 10.8 ± 3.2 | <.001 |
| HOMA-IR | 1.7 ± 0.6 | 1.9 ± 0.7 | 2.1 ± 0.8 | 2.3 ± 0.8 | <.001 |

Abbreviations: BMI, body mass index; WC, waist circumference; WHR, waist-to-hip ratio; WHtR, waist-to-height ratio; SFT, skin-fold thickness; SBP, systolic blood pressure; DBP, diastolic blood pressure; AST, aspartate aminotransferase; ALT, alanine aminotransferase; HOMA-IR, Homeostasis model assessment insulin resistance. *P-values were adjusted for sex and age (except for the trend of age itself). **Percent body fat was measured for 541 adolescents (277 males and 264 females).
