# Supplementary material for: Association between Obesity Indices and Insulin Resistance among Healthy Korean Adolescents: The JS High School Study
Source: PLoS One. 2015 May 13;10(5):e0125238. doi: 10.1371/journal.pone.0125238 (PMC4429969; doi:10.1371/journal.pone.0125238)
Supplement: S4 Table — (DOCX) [file pone.0125238.s004.docx]

S4 Table. Risk for Insulin Resistance by Different Obesity Indices in Male and Female Adolescents

| Independent variable  (obesity index) | Male | | | |  | Female | | | |
| --- | --- | --- | --- | --- | --- | --- | --- | --- | --- |
|  | No. of people | HOMA-IR >2.5 | Age-adjusted OR  (95% CI) | |  | No. of people | HOMA-IR >2.5 | Age-adjusted OR (95% CI) | |
| Weight-for-height percentile |  |  |  |  |  |  |  |  |  |
| <50 | 196 | 16 | 1.00 |  |  | 167 | 18 | 1.00 |  |
| 50-74 | 106 | 11 | 1.32 | (0.59 - 2.97) |  | 122 | 17 | 1.37 | (0.67 - 2.79) |
| 75-94 | 90 | 27 | 4.82 | (2.43 - 9.57) |  | 85 | 22 | 3.04 | (1.52 - 6.09) |
| ≥95 | 26 | 18 | 26.08 | (9.71 - 70.00) |  | 25 | 9 | 4.89 | (1.87 - 12.78) |
| BMI-for-age percentile |  |  |  |  |  |  |  |  |  |
| <50 | 178 | 14 | 1.00 |  |  | 182 | 22 | 1.00 |  |
| 50-74 | 111 | 12 | 1.40 | (0.62 - 3.15) |  | 114 | 14 | 1.03 | (0.50 - 2.11) |
| 75-94 | 101 | 27 | 4.21 | (2.08 - 8.52) |  | 81 | 21 | 2.63 | (1.34 - 5.14) |
| ≥95 | 28 | 19 | 24.71 | (9.37 - 65.18) |  | 22 | 9 | 5.22 | (1.99 - 13.72) |
| WC-for-age percentile |  |  |  |  |  |  |  |  |  |
| <50 | 201 | 17 | 1.00 |  |  | 132 | 11 | 1.00 |  |
| 50-74 | 129 | 17 | 1.65 | (0.81 - 3.37) |  | 126 | 17 | 1.69 | (0.76 - 3.78) |
| 75-94 | 73 | 28 | 6.67 | (3.34 - 13.28) |  | 111 | 25 | 3.15 | (1.47 - 6.74) |
| ≥95 | 15 | 10 | 22.62 | (6.84 - 74.77) |  | 30 | 13 | 8.62 | (3.32 - 22.39) |
| WHR percentile |  |  |  |  |  |  |  |  |  |
| <50 | 208 | 20 | 1.00 |  |  | 199 | 18 | 1.00 |  |
| 50-74 | 105 | 11 | 1.11 | (0.51 - 2.41) |  | 100 | 19 | 2.39 | (1.19 - 4.80) |
| 75-94 | 84 | 27 | 4.49 | (2.34 - 8.61) |  | 79 | 18 | 2.89 | (1.41 - 5.93) |
| ≥95 | 21 | 14 | 16.79 | (5.97 - 47.21) |  | 21 | 11 | 11.13 | (4.15 - 29.88) |
| WHtR percentile |  |  |  |  |  |  |  |  |  |
| <50 | 209 | 17 | 1.00 |  |  | 199 | 21 | 1.00 |  |
| 50-74 | 104 | 12 | 1.56 | (0.71 - 3.42) |  | 101 | 13 | 1.25 | (0.60 - 2.61) |
| 75-94 | 85 | 30 | 6.31 | (3.22 - 12.34) |  | 80 | 23 | 3.59 | (1.84 - 7.01) |
| ≥95 | 20 | 13 | 20.85 | (7.27 - 59.80) |  | 19 | 9 | 7.93 | (2.87 - 21.94) |
| SFT-for-age percentile |  |  |  |  |  |  |  |  |  |
| <50 | 190 | 15 | 1.00 |  |  | 164 | 24 | 1.00 |  |
| 50-74 | 122 | 17 | 2.10 | (1.00 - 4.43) |  | 116 | 18 | 1.11 | (0.57 - 2.16) |
| 75-94 | 87 | 28 | 5.65 | (2.80 - 11.38) |  | 84 | 13 | 1.10 | (0.53 - 2.29) |
| ≥95 | 18 | 11 | 22.48 | (7.33 - 68.90) |  | 35 | 11 | 2.89 | (1.24 - 6.72) |
| Percent body fat percentile* |  |  |  |  |  |  |  |  |  |
| <50 | 136 | 16 | 1.00 |  |  | 130 | 21 | 1.00 |  |
| 50-74 | 69 | 11 | 1.43 | (0.62 - 3.27) |  | 67 | 18 | 1.85 | (0.90 - 3.78) |
| 75-94 | 57 | 16 | 2.93 | (1.35 - 6.39) |  | 53 | 14 | 1.80 | (0.83 - 3.90) |
| ≥95 | 15 | 13 | 47.58 | (9.81 - 230.8) |  | 14 | 5 | 2.93 | (0.89 - 9.66) |
| One SD increase in |  |  |  |  |  |  |  |  |  |
| Body weight | 418 | 72 | 2.35 | (1.80 - 3.07) |  | 399 | 66 | 1.60 | (1.24 - 2.06) |
| BMI | 418 | 72 | 2.74 | (2.07 - 3.62) |  | 399 | 66 | 1.69 | (1.31 - 2.18) |
| WC | 418 | 72 | 2.56 | (1.96 - 3.35) |  | 399 | 66 | 1.93 | (1.48 - 2.51) |
| WHR | 418 | 72 | 2.36 | (1.80 - 3.11) |  | 399 | 66 | 1.97 | (1.49 - 2.59) |
| WHtR | 418 | 72 | 2.73 | (2.07 - 3.61) |  | 399 | 66 | 1.96 | (1.50 - 2.55) |
| SFT | 417 | 71 | 2.54 | (1.92 - 3.36) |  | 399 | 66 | 1.33 | (1.03 - 1.71) |
| Percent body fat | 277 | 56 | 2.68 | (1.92 - 3.74) |  | 264 | 58 | 0.96 | (0.72 - 1.30) |

Abbreviations: BMI, body mass index; WC, waist circumference; WHR, waist-to-hip ratio; WHtR, waist-to-height ratio; SFT, skin-fold thickness; HOMA-IR, Homeostasis model assessment insulin resistance. *Percent body fat was measured for 541 adolescents (277 males and 264 females).
